# Adaptation and altitude sickness: A 40-year bibliometric analysis and collaborative networks

**DOI:** 10.3389/fpubh.2023.1069212

**Published:** 2023-03-02

**Authors:** J. Pierre Zila-Velasque, Pamela Grados-Espinoza, Cristian Morán-Mariños, Kevin O. Morales Pocco, Uriel S. Capcha-Jimenez, Zhamanda N. Ortiz-Benique

**Affiliations:** ^1^Red Latinoamericana de Medicina en la Altitud e Investigación, Pasco, Peru; ^2^Facultad de Medicina Humana, Universidad Nacional Daniel Alcides Carrión, Pasco, Peru; ^3^Unidad de Investigación en Bibliometría, Vicerrectorado de Investigación, Universidad San Ignacio de Loyola, Lima, Peru; ^4^Servicio de Neumología, Hospital Nacional Dos de Mayo, Lima, Peru; ^5^Red de Eficacia Clinica y Sanitaria (REDECS), Lima, Peru; ^6^Universidad Nacional del Altiplano Puno, Puno, Peru; ^7^Asociación Científica de Estudiantes de Medicina – UNAP, Puno, Peru; ^8^Facultad de Medicina, Universidad Nacional Mayor de San Marcos, Lima, Peru; ^9^Sociedad Científica de San Fernando, Lima, Peru; ^10^Facultad de Medicina, Universidad Nacional de San Agustín, Arequipa, Peru

**Keywords:** altitude sickness, acclimatization, altitude, bibliometrics, adaptation, hypoxia

## Abstract

**Introduction:**

We analyze the scientific production and collaboration networks of studies based on adaptation and altitude diseases in the period 1980–2020.

**Methods:**

The publications were extracted from journals indexed in Scopus. The bibliometric analysis was used to analyze the scientific production, including the number of annual publications, the documents, and the characteristics of the publications. With the VOSviewer software, the analysis of collaborative networks, productivity of the countries, as well as the analysis of the co-occurrence of keywords were visualized.

**Results:**

15,240 documents were registered, of which 3,985 documents were analyzed. A significant trend was observed in the number of publications (*R*^2^: 0.9847; *P*: < 0.001), with annual growth of 4.6%. The largest number of publications were original articles (77.8%), these published more frequently in the journal “Altitude Medicine and Biology”. The largest number of countries were from Europe and Asia; however, the largest collaboration network was with the United States. Of the countries with high altitudes, China and Peru ranked first in scientific productivity. The research priorities were on the adaptation mechanism (37.1%), mainly anoxia and respiratory function. Acute mountain sickness (18.4%) and pulmonary edema (14.7%) were the most reported diseases. Of the top 10 institutions, “University of Colorado” and “Universidad Peruana Cayetano Heredia” contributed more than 100 publications.

**Conclusions:**

Scientific production on adaptation and altitude illnesses continues to grow. The United States and United Kingdom present collaborative networks with high-altitude countries. The research is aimed at studying the mechanisms of adaptation to altitude and acute mountain sickness.

## Introduction

Altitude diseases are presented in relation to the speed of ascent with respect to meters above sea level (m.a.s.l.), adaptability and acclimatization. A prevalence between 40 and 90% is reported according to altitude (1), and an incidence between 57 and 73.5% as it happens in Tibet, and Everest, respectively (2, 3). This occurs much less frequently in the Andean population or in Tibetan ethnic groups, which can be attributed to physiological and genetic adaptation (4–7).

Given the complexity of the development of these diseases and the adaptability relationships, it has been possible to generate multiple publications that have contributed to their understanding, such as sleep disorders (8), simulation tests in hypoxic states (9), physiological and genetic considerations in age groups (10, 11), treatments (12), and epidemiological studies (13, 14). However, the only study that we observed denotes a scarce production in the scientific production on this area (15).

Bibliometric studies are a method that allows scientific productivity to be measured in multiple fields, with this it will be possible to identify the pattern of published studies, the geographical distribution and the network of scientific collaboration that they generated between the countries and allowed the knowledge of various diseases such as the epilepsy or lupus (16, 17).

Given that high altitude medicine research may show greater interest in altitude diseases, as well as knowledge about the health status and quality of life of these people, we consider it vitally important to characterize their evolution in this field. Therefore, this study aims to carry out a bibliometric analysis, evaluate the scientific production on adaptation studies and altitude-related diseases, and collaborative networks, which allow us to address these issues, highlighting emerging issues and gaps in knowledge.

## Methods and materials

A search strategy (SS) of documents published between 1980 and 2020 of journals indexed to Scopus in November 2021 was carried out.

### Source of information

This base was selected for its eligibility of quality journals and multidisciplinary topics (18). It also retrieves a greater number of indexed journals, contains more information available by giving 100% coverage to Medline/Embase, and features more content from non-North American sources compared to Web of Science and Pubmed. Finally, it allows the generation of bibliometric indicators from its own analysis system that is complemented with indicators from SCImago Journal Rank (SJR) and Scopus (19).

### Search strategy

A search strategy (SS) was built from terms of Medical Subject Headings - MeSH (Pubmed) and emtree terms (Embase).

- SS #1: Includes general words such as “Altitude Sickness,” “Altitude Sickness,” “Acclimatization”. These words were coupled with complications of maladaptation or altitude sickness, these included: Ventilatory or hemoglobin or “maximum sleeping” or cardiovascular or cardiac or pulmonary^*^ or lung or cerebral or kidney, etc.
- SS # 2: SS # 1 was limited to field codes for subject areas in Scopus. SUBJAREA (medi OR nurses OR dent OR cure).

**Figure 1:**
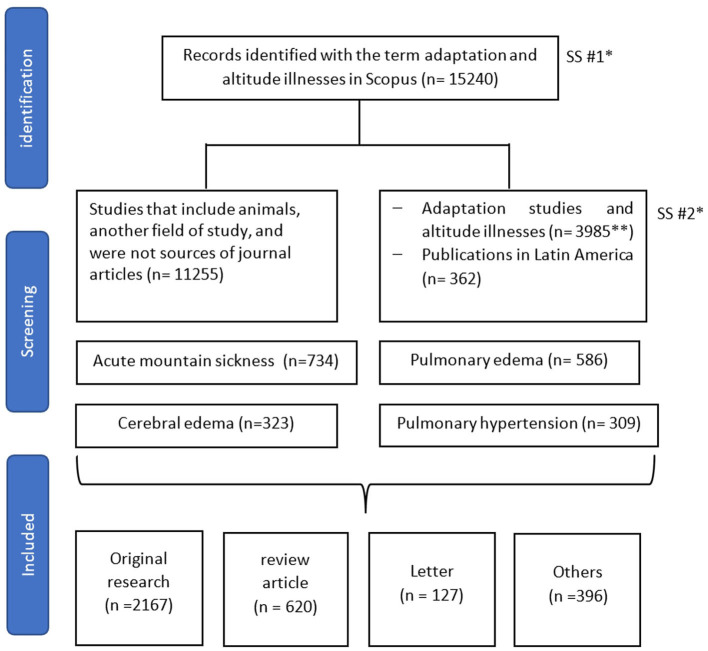
Flowchart of study selection. *SS: Search strategy; **Medicine, Nursing, Dentistry, Health professions.

The final SS were limited to publications to a journal source type (SRCTYPE, “j”) and keywords such as “rats” or “animal^*^” were excluded. Figure 1 shows the flowchart of the bibliometric search. The search strategy is provided as Supplementary material 1.

### Analysis of data

The records were exported by default to Microsoft Excel 2021. The analysis was divided into two stages: (1) Analysis of data between 1980 and 2020 with VOSviewer, and (2) analysis of data between 2012 and 2020 with SciVal.

–In the first data analysis, data was determined in: (1) annual publications (compared to Scielo) and type of publications, (2) Scientific production by institutions and journals with their respective metrics available in SCImago & Scopus. Other results are available as Supplementary material 2, these include the top 10 most prolific publications, institutions, and their research departments with the longest track record, and the top 50 journals with the largest number of publications.

The bibliometric indicators allow us to know a general classification of the journals (quartiles, Q1–Q4), consider the citations according to their importance (SJR) and offer a more complete and transparent vision of their impact (CiteScore) (20).

The results were presented in tables and graphs of frequency and percentage. Likewise, the Pearson correlation was applied to better understand the trends of the publications and the Student's *t*-test to determine the differences in the number of publications between ranges of years. A *p*-value < 0.05 was considered significant.

Two maps were generated from VOSviewer version 1.6.6 software (Leiden University, Leiden, The Netherlands). (1) Map of scientific collaboration between countries. (2) Co-occurrence map of keywords from studies based on adaptation and altitude illnesses. Its graphic interpretation is based on the size of the circle, thickness of the connection and distance between elements (countries or keywords). The size of the circle will be proportional to the number of documents or total occurrences; the color will depend on the related cluster group and the thickness of the line will be the link strength between the elements. Additionally, thesaurus methods were applied to give the maps greater representativeness (21).

The second analysis (2017–2020) used SciVal, it is an online tool (Elsevier) based on the Scopus database that complements research metrics (22). The generated results included analysis in collaboration networks, authorship, topics, research areas (25% of the most important topics worldwide in adaptation and altitude diseases). The results are available as Supplementary material 3.

### Ethics

Data were downloaded from available published research; therefore, ethical approval was not required.

## Results

### Evidence summary

Our initial search resulted in 15,240 papers on adaptation studies and altitude sickness. In the delimitation in health issues (SS#2), 3,985 documents were obtained that were analyzed in our study, of these 362 were from Latin America (Figure 1).

### Trends in scientific production

**Figure 2:**
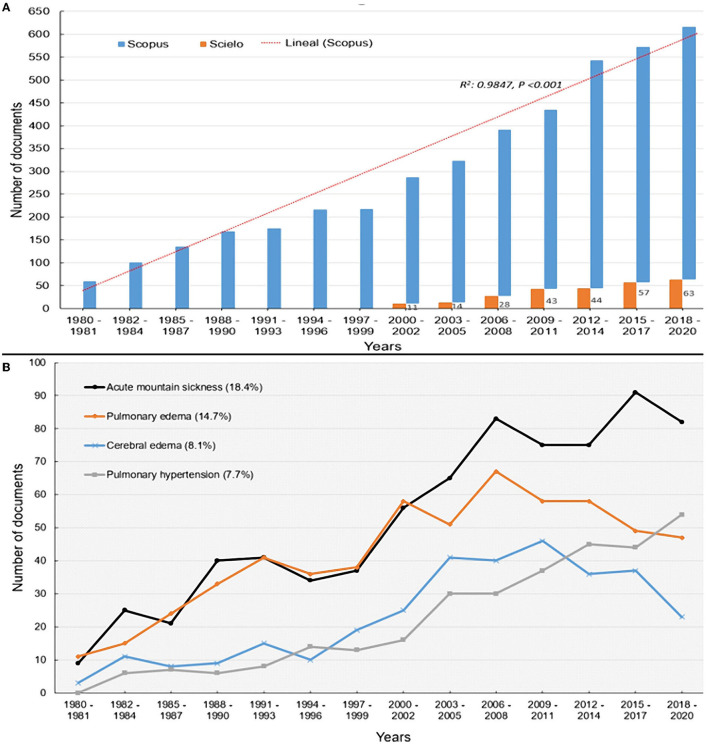
Trend of scientific production on adaptation and altitude illnesses, 1980–2020. **(A)** Trend of publications per year. **(B)** Record of diseases and complications due to high-altitude.

An increasing trend is observed with a very strong and significant correlation (*R*^2^: 0.984; *P*: < 0.001) between the number and publication date. The annual average was 97 documents with a growth rate of 4.6% per year. The highest number of investigations on diseases at altitude were acute mountain sickness (18.4%), pulmonary edema (14.7%), cerebral edema (8.1%) and pulmonary hypertension (7.7%) (Figure 2).

In the last two decades 2001–2020 there was a significant increase of up to 2.5 times greater in scientific production (*p* = 0.05) compared to 1980 and 2000. Most of the publications were original articles (*n* = 3,103; 77.8%) and reviews (*n* = 497; 12.5%) (Table 1).

**Table 1:** Years of publication and types of journal articles.

**Years**	**Documents**	**Original research**	**Review articles**	**Letter**	Other^*^	***p* ^a^**
1980–1990	467	409	19	23	16	0.05 Mean diff. (824)
1991–2000	701	555	60	20	66	
2001–2010	1,120	827	174	34	85	
2011–2020	1,697	1,312	244	57	84	
Total	3,985	3,103 (77.8%)	497 (12.4%)	134 (3.3%)	251 (6.2%)	

* Others: Conference Paper, Note, Editorial, Short Survey, Erratum.

a Significant *p* between (1980–2000) and (2001–2020).

### Top 10 journals with the highest scientific production

The top 10 journals with the highest publication record had a minimum of 34 papers between 1980 and 2020. The top journals included “High-altitude Medicine and Biology” with 388 (8.5%) publications, followed by “Journal of Applied Physiology” 155(3.9 %) and “Wilderness and Environmental” 132 (3.3%). In the classification of journals: 6 journals belonged to Quartile Score (quartile Q1), 3 (Q2) and 1 (Q3).The journal “Journal of Applied Physiology” was the one that obtained the highest number of citations (7,937), while “New England Journal of Medicine” was the one that received the highest number of citations for each document, it also presents the highest CiteScore (110.5) and SJR (24.907). found a high rate of collaboration in the journal with objectives and scope in high-altitude medicine in more than 30% (Table 2).

**Table 2:** Top 10 journals with the most articles published on research at altitude in Scopus, 1980–2020.

**Journals**	**Number of articles (%)**	**Quartile scores**	**Number of citations**	**Citations/ documents**	**CiteScore^a^**	**SJR^a^**	**% international collaboration^b^**
High-altitude Medicine and Biology	338 (8.5)	Q2	6,568	19.4	3.9	0.695	37.7
Journal of Applied Physiology	155 (3.9)	Q1	7,937	51.2	5.9	1.003	40.2
Wilderness and Environmental Medicine	132 (3.3)	Q2	1,769	13.4	2.4	0.399	15.7
Aerospace Medicine and Human Performance	111 (2.8)	Q3	2,343	21,1	1.3	0.265	11.2
Chest	49 (1.2)	Q1	1,741	35.5	10	2.264	20.9
International Journal of Sports Medicine	49 (1.2)	Q1	1,697	34.6	4.5	0.971	33.1
Respiratory Physiology and Neurobiology	44 (1.1)	Q2	1,085	24.7	4	0.635	25.8
European Respiratory Journal	37 (0.9)	Q1	1,199	32.4	18.9	4.336	45.1
Frontiers in Physiology	37 (0.9)	Q1	441	11.9	6.6	1.126	34.8
New England Journal of Medicine	34 (0.8)	Q1	3,453	101,6	110.5	24.907	22.5

a Scopus (2021).

b Scimago Journal and Country Rank (2021).

The first 50 scientific journals with the most publications on adaptation and altitude illnesses were frequently from countries such as the United States and United Kingdom, were presented with more than 7 publications and the journal “Journal of Applied Physiology” was the one that obtained the highest number of citations (Table 3 in Supplementary material 2). No Latin American, Asian, or African journals were found.

### Co-occurrence of countries and keywords

**Figure 3:**
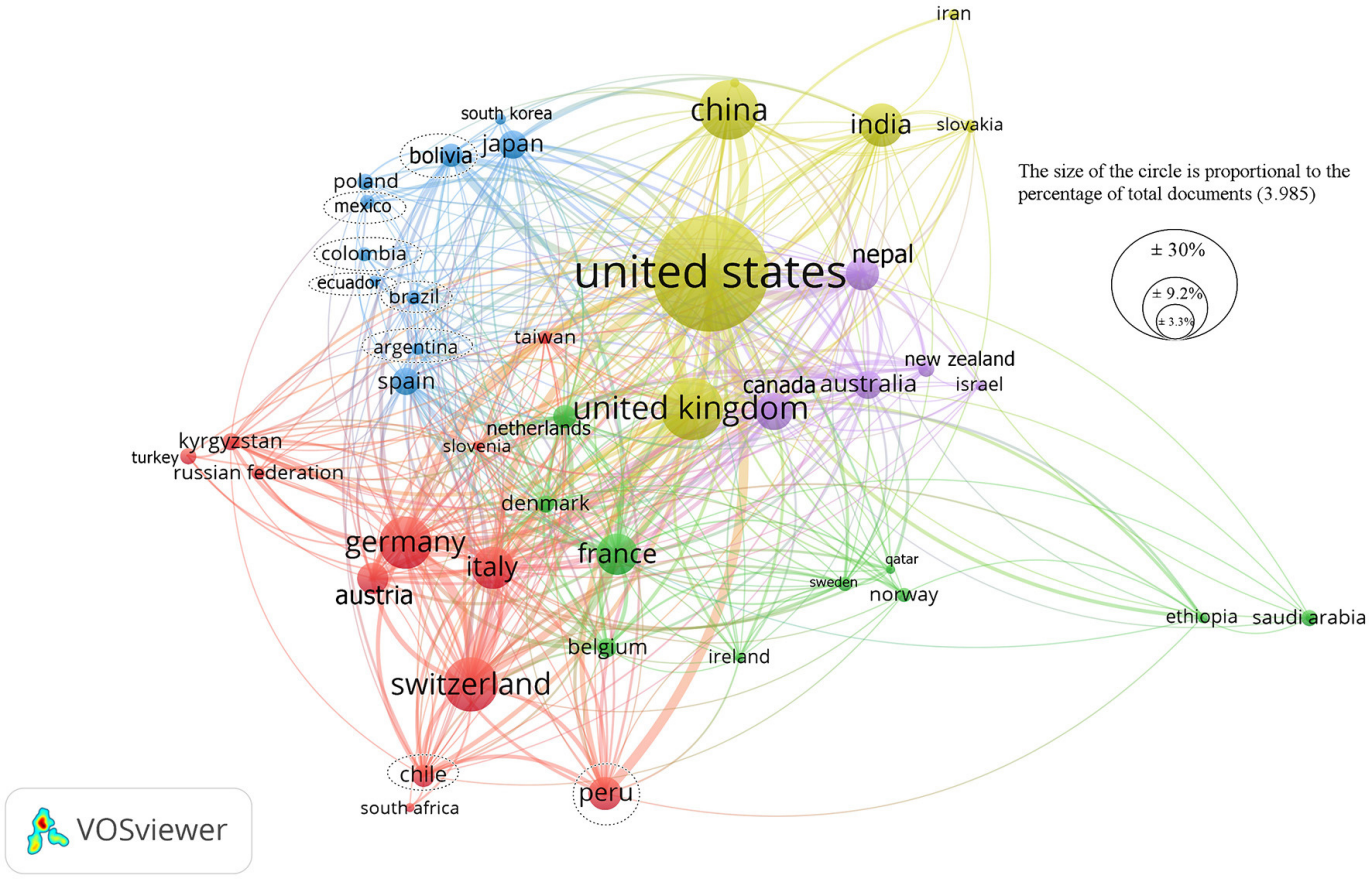
Worldwide scientific collaboration network between countries on adaptation and altitude illnesses. The dotted lines indicate the countries of Latin America.

Forty-two of 180 countries were registered, which had a minimum of 10 publications. The largest participation was from the continent of Europe with 16 countries, followed by Asia (11) and Latin America (8). The countries with the largest collaborative networks and scientific production worldwide were the United States with 40 countries, followed by the United Kingdom (37), Germany (34), Switzerland and Canada (33). The countries with the highest altitude (cities with an average altitude > 2,500 m.a.s.l) and the number of publications are: China, with a total of 363 publications, followed by Peru (130), Nepal (128), Bolivia (70) and Colombia (29) (Figure 3).

**Figure 4:**
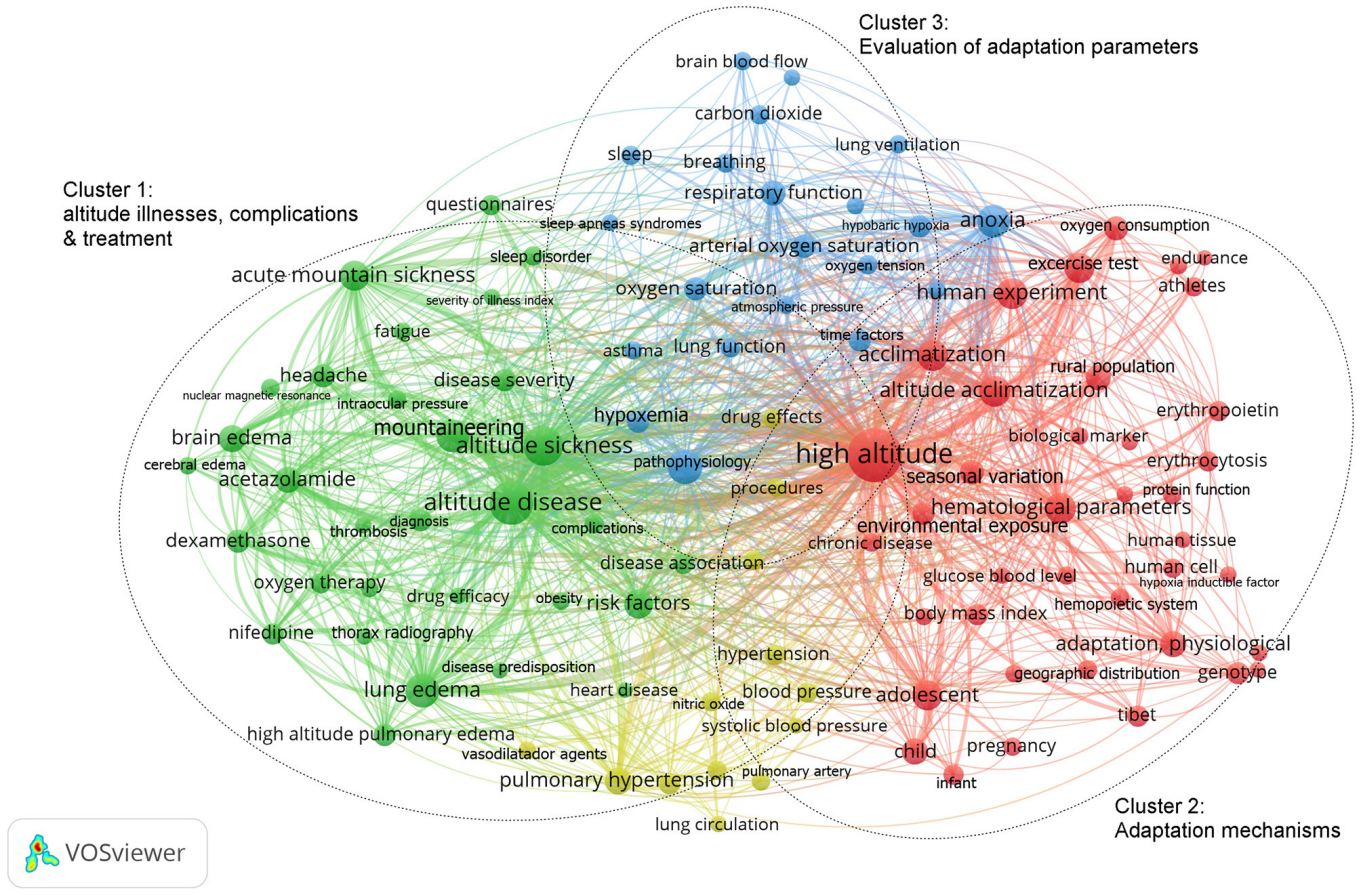
Network of occurrences of the map of keywords in adaptation and altitude illnesses.

There were 104 of 15,910 keywords that were used in 3,985 documents that had a minimum of 50 occurrences. These were then arranged and presented in groups as determined by the total number of occurrences. Group 2: Adaptation mechanisms (10,119 cases; 37.1%); Group 3: Adaptation parameters (8,889 cases; 32.6%); Group 1: Altitude illnesses, complications, and treatment (8,289; 30.4%). Likewise, within the groups, keywords were identified, with a greater number of occurrences. Group 3: hypoxia, acclimatization, and metabolism. Group 2: anoxia, exercise test and respiratory function. Group 1: mountain acute sickness, lung edema and pulmonary hypertension (disease and complication); acetazolamide dexamethasone and nifedipine (treatment) (Figure 4).

Between 2012 and 2020, publications on “acute lung edema” and “acetazolamide” represented the topic with the highest number of publications (Top 25% of world topics by prominence). However, for 2020, the topics in “COVID-19,” “Radiological finding,” and “clinical features” was the one that received the highest priority or scientific impulse (Figure 2 in Supplementary material 3).

### Publications and institutional collaboration

The largest number of institutions were from the United States (4/10) and Switzerland (3/10); Peru, Germany and Nepal only had one institution. “University of Colorado” obtained the highest publication number (n: 219), followed by “Universidad Peruana Cayetano Heredia” (n: 101). The highest number of document citations was from “University of Colorado.” The highest percentage of publications with affiliation from a country was from the “United States Army Research Institute of Environmental Medicine”, while “University of Colorado” obtained the highest number of collaborations with other countries (Table 3).

**Table 3:** Top 10 publications and institutional collaboration on adaptation and altitude illnesses in Scopus, 1980–2020.

**Institution (country)**	**Total documents**	**Total citation**	**Citation/ documents**	**National (%)**	**International (%)**
- University of Colorado (*United States*)	282	1,591	56.4	192 (68.1)	90 (31.9)
- Universidad Peruana Cayetano Heredia (*Peru*)	101	2,420	24	35 (34.7)	66 (65.3)
- University of Washington (*United States*)	83	4,420	53,3	58 (69.9)	25 (30.1)
- UniversitatsSpital Zurich (*Switzerland*)	83	3,797	45,7	35 (42.2)	48 (57.8)
- University of California, San Diego (*United States*)	69	2,387	34,6	49 (71)	20 (29)
- Universitätsklinikum Heidelberg (*Germany*)	56	2,099	37,5	27 (48.2)	29 (51.8)
- University of Zurich (*Switzerland*)	53	1,398	26,4	30 (56.6)	23 (43.4)
- United States Army Research Institute of Environmental Medicine (*United States*)	49	1,947	39,7	46 (93.9)	3 (6.1)
- Nepal International Clinic (*Nepal*)	49	1,106	22,6	22 (44.9)	27 (55.1)
- University Hospital Bern (*Switzerland*)	48	1,871	38,9	30 (62.5)	18 (37.5)

With the VOSviewer tool we determined that Peru and Switzerland are the countries that have different departments or areas that are within the same institution that investigates adaptation and altitude diseases (Table 2 in Supplementary material 2).

## Discussion

Our study showed an increase of up to 230% in the scientific production on adaptation and altitude diseases worldwide from 2001 to 2020. This would be possible due to the prevalence of these diseases and the participation of different specialties of medicine, areas not medical centers and centers specializing in altitude illnesses. It can be demonstrated by determining the scientific production of 2021, which obtained 242 documents. However, this increase may be due to studies on COVID-19 during the pandemic, which appears as the main topic in adaptation at height (Figure 2 in Supplementary material 3) and other areas (23–25).

The United States, the United Kingdom and China covered around 48% of the total scientific production. The possible determinants would be that high-income countries have expenses in research and development (% of GDP) and promote competitiveness, likewise, they seek to achieve higher levels of development and scientific cooperation (26, 27). However, we consider that the geographical location and the number of population to study (cities > 2,500 m.a.s.l) influence the number of publications and allowed countries such as Peru, Nepal, Bolivia and Colombia to reduce the gap between the number of publications of countries high and low income (28). In a similar way, it could be explained with prevalent diseases in a certain geographical area (29). Therefore, we hope that soon the percentage of international collaboration (33.4%) will be higher in the coming years (Table 2 in Supplementary material 3).

Studies on diseases and adaptability at altitude not only involve more complex methods but also the mobilization of researchers to certain geographical areas (30, 31). This allows for common benefit such as access to scarcely available resources, improving scientific quality and strengthening international cooperation (31–33). With this, a greater scientific merit is achieved with the type of publication in original article or review and higher quality journals (CiteScore & SJR) (20). This would motivate the main authors to publish in journals such as “New England Journal of Medicine” and “European Respiratory Journal”, however, these journals, due to their more limited objectives and scope, would explain a lower number of publications compared to “High-altitude Medicine and Biology” that present objectives, not only focused on medical sciences, but also biology, anthropology, and human ecology.

It was recorded that four of the ten institutions with the most publications come from the United States, this is mainly supported by financing and amount of resources as explained above. However, the countries of Peru and Nepal stand out (Table 2 in Supplementary material 2). This achievement is confirmed by the research centers they present and the use of their geographical location. Particularly in Peru, the “Universidad Peruana Cayetano Heredia” through the “Instituto de Investigaciones de la Altura” and the research unit of the “Centro Latinoamericano de Excelencia en Cambio Climático y Salud” would explain its scientific production comparable to other institutions in developed countries (34). We believe that the countries of Asia and in particular of Latin America can develop larger research centers and inter-institutional agreements, since this would favor people who live in Andean regions and represent up to 44% of the total population of each country (35, 36).

We mainly observe acute mountain sickness, pulmonary and cerebral edema as the most prolific; it is most likely due to mortality, complications and the treatment that it would imply as visualized in the VOSviewer graph (Figure 4). However, it is also necessary to recognize the impact or burden of other diseases that living at altitude would generate in relation to respiratory diseases such as infectious and chronic diseases such as COPD or pulmonary hypertension, since studies are still being published that update the concepts on survival or physiological adaptations (37–39). We hope that this can provide the impetus for future research. Finally, we believe that other fields in high altitude medicine should be explored, this is because there are medical specialties such as surgery, emergency medicine, and pediatrics that are beginning to show interest in this area (Figure 1 in Supplementary material 3). We hope that this can provide the impetus for future research.

Finally, one of the limitations of bibliometric studies is the use of a single database, which does not allow the evaluation of articles that have been published in other databases. However, it shows a similar trend, but to a lesser extent when using the Scielo database; this database is used by authors from Latin America. Another limitation is the search strategy, this is due to the possibility of overestimating the value obtained from a real value when using keywords that Scopus can recognize from topics not related to our objective. Despite the above, we believe that the SS we make is of high quality and can be replicated in other studies.

## Conclusion

Scientific production on adaptation and altitude illnesses is constantly growing. The United States, the United Kingdom and Germany were the ones that published the most, these present collaborative networks with some high-altitude countries. More studies on adaptation mechanisms are currently being published and prioritize diseases such as acute mountain sickness and pulmonary edema, however, it is necessary to explore other areas. Having a solid institution with financial support allowed the Universidad Peruana Cayetano Heredia to reach a global level.

## Data availability statement

The original contributions presented in the study are included in the article/Supplementary material, further inquiries can be directed to the corresponding author.

## Author contributions

JZ-V and PG-E: conceptualization and supervision. CM-M and JZ-V: data curation and methodology. CM-M: formal analysis, resources, software, and visualization. JZ-V, PG-E, KM, UC-J, and ZO-B: investigation and writing—original draft. JZ-V: project administration and validation. JZ-V, PG-E, CM-M, KM, UC-J, and ZO-B: writing—review and editing. All authors contributed to the article and approved the submitted version.
