# Supplementary material for: Adaptation and altitude sickness: A 40-year bibliometric analysis and collaborative networks
Source: Front Public Health. 2023 Mar 2;11:1069212. doi: 10.3389/fpubh.2023.1069212 (PMC10018125; doi:10.3389/fpubh.2023.1069212)
Supplement: Supplementary file 1 [file Data_Sheet_1.docx]

**Supplementary 1.** Search strategy

| Database | | | Results* |
| --- | --- | --- | --- |
| Scopus | | | |
| SS#1 | #1 | TITLE-ABS(Adaptatio* or adaptive or plasticity or acclimatization) OR TITLE-ABS(Adaptation w/2 genetic) OR TITLE-ABS(Adaptation w/2 physiologic*) or TITLE-ABS(Adaptation w/2 plasticity) or TITLE-ABS(Adaptation w/2 behavior*) OR TITLE-ABS(Adaptation w/2 Phenotypic) | 3985 |
|  | #2 | TITLE-ABS-KEY(Altitud* w/4 high*) or TITLE-ABS-KEY(Altitud* w/5 mountain) OR TITLE-ABS-KEY(altitud* w/5 hypoxia) |  |
|  | #3 | #1 AND #2 |  |
|  | #4 | TITLE-ABS(Ventilatory or hemoglobin or “máximum sleeping” or cardiovascular or cardiac or pulmonar* or lung or cerebral or kidney or blood or saturation or sleep* or edema or disease or illnesses or acute or prevention or drug* or treatment* or medicine or “covid-19” or "SARS-COV-2") |  |
|  | #5 | #2 AND #4 |  |
|  | #6 | #3 OR #5 |  |
| SS#2 | #7 | SUBJAREA(MEDI OR NURS OR DENT OR HEAL) |  |
|  | #8 | #6 AND #7 |  |
|  | #9 | TITLE-ABS-KEY(Animal* or rat* or mice) |  |
|  | #10 | #8 AND NOT #9 |  |
|  | #11 | ( PUBYEAR > 1979 ) AND ( LIMIT-TO ( SRCTYPE,"j" ) ) AND EXCLUDE ( PUBYEAR,2021) AND EXCLUDE (PUBYEAR,2022) |  |
|  | #12 | #10 AND #11 |  |
| Scielo | | | |
| #1 | | TS=(Adaptatio* or adaptive or plasticity or acclimatization) OR TS=(Adaptation NEAR/2 genetic) OR TS=(Adaptation NEAR/2 physiologic*) OR TS=(Adaptation w/2 plasticity) OR TS=(Adaptation NEAR/2 behavior*) OR TS=(Adaptation NEAR/2 Phenotypic) OR TS=(Adaptaci* or aclimatizacion) | 264 |
| #2 | | TS=(Altitud* NEAR/4 high*) or TS=(Altitud* NEAR/5 mountain) OR TS=(altitud* NEAR/5 hypoxia) |  |
| #3 | | #1 AND #2 |  |
| #4 | | TS=(Ventilatory or hemoglobin or “máximum sleeping” or cardiovascular or cardiac or pulmonar* or lung or cerebral or kidney or blood or saturation or sleep* or edema or disease or illnesses or acute or prevention or drug* or treatment* or medicine or “covid-19” or "SARS-COV-2") |  |
| #5 | | #2 AND #4 |  |
| #6 | | #3 OR #5 |  |
| #7 | | (TS=(“edema cerebral de altura” or "mal agudo de montaña" or “enfermedad de altura” or “mal de altura”) or TS=(“gran altitud” NEAR/4 enfermedad)) |  |
| #8 | | #6 or #7 |  |

*Until December 2021
