# Supplementary material for: Adaptation and altitude sickness: A 40-year bibliometric analysis and collaborative networks
Source: Front Public Health. 2023 Mar 2;11:1069212. doi: 10.3389/fpubh.2023.1069212 (PMC10018125; doi:10.3389/fpubh.2023.1069212)
Supplement: Supplementary file 2 [file Data_Sheet_2.pdf]

Supplementary material 2

Table 1. Top 10 publications with the most citations in research on adaptation & diseases at altitude in Scopus, 1980 - 2020

Rank	First uthors	Article titule	Article type	Scopus source title	Year	Total citations	Average citations per year
1	Hackett P.H. (2001)	High-altitude illness	Review	New England Journal of Medicine	2001	940	49.4
2	Levine B.D. (1997)	Living high-training low': Effect of moderate-altitude acclimatization with low-altitude training on performance	Article	Journal of Applied Physiology	1997	525	22.8
3	Sylvester J.T. (2012)	Hypoxic pulmonary vasoconstriction	Article	Physiological Reviews	2012	398	49.8
4	Bigham A. (2010)	Identifying signatures of natural selection in Tibetan and Andean populations using dense genome scan data	Article	PLoS Genetics	2010	373	37.3
5	Basnyat B. (2003)	High-altitude illness	Conference Paper	Lancet	2003	366	21.5
6	Maggiorini M. (2001)	High-altitude pulmonary edema is initially caused by an increase in capillary pressure	Article	Circulation	2001	331	17.4
7	Haase V.H. (2013)	Regulation of erythropoiesis by hypoxia-inducible factors	Article	Blood Reviews	2013	329	47.0
8	Hartmann G. (2000)	High altitude increases circulating interleukin-6, interleukin-1 receptor antagonist and C-reactive protein	Article	Cytokine	2000	315	15.8
9	León-Velarde F. (314)	Consensus statement on chronic and subacute high-altitude diseases	Review	High Altitude Medicine and Biology	2005	314	20.9
10	Bartsch P. (1991)	Prevention of High-Altitude Pulmonary Edema by Nifedipine	Article	New England Journal of Medicine	1991	312	10.8

Table 2. Most prolific institutions in research on adaptation and altitude diseases, 1980 - 2020

Country - Institutions	Documents¥	Citations	total link strength¥
**Nepal**			
Himalayan rescue association, kathmandu.	23	1004	20
Nepal international clinic, kathmandu.	18	663	14
Mountain medicine society of nepal, kathmandu.	15	193	20
**United states**			
Department of anthropology, case western reserve university, cleveland, oh.	17	921	8
Institute for altitude medicine, telluride, co	11	520	7
Division of pulmonary and critical care medicine, university of Washington, nseattle, wa.	11	381	2
**Peru**			
Universidad peruana cayetano heredia, lima, peru*	22	274	8
Cronicas center of excellence in chronic diseases, universidad peruana cayetano heredia, lima.			
Departamento de ciencias biológicas y fisiológicas, universidad peruana cayetano heredia, lima.			
Departamento de medicina, escuela de medicina, universidad peruana cayetano heredia, lima.			
Instituto de investigaciones de la altura, universidad peruana cayetano heredia, lima.	Most publications and citations		
**United kingdom**			
Department of physiology, anatomy and genetics, university of oxford, oxford.	6	322	0
Department of physiology, development and neuroscience, university of cambridge, cambridge.	6	257	2
Medical school, university of birmingham, birmingham.	5	225	2

Country - Institutions	Documents¥	Citations	total link strength¥
**Australia**			
Department of medicine, university of sydney, sydney, nsw.	6	151	4
Department of physiology, australian institute of sport, canberra.	6	201	6
Exercise physiology laboratory, flinders university, adelaide.	5	223	8
**Switzerland**			
University of zurich, zurich*	17	417	7
Institute of physiology, university of zurich, zurich.	Most publications and citations		
Center for integrative human physiology, university of zurich, zurich.			
Institute of veterinary physiology, vetsuisse faculty, university of zurich, zurich.			
Zurich center for integrative human physiology, university of zurich, zurich.			
**Germany**			
Department of sports medicine/sports physiology, university of bayreuth, bayreuth.	10	747	10
**kyrgyzstan**			
Institute of molecular biology and medicine, bishkek.	5	221	4
National center of cardiology and internal medicine, bishkek.	5	161	4
**Chile**			
Facultad de ciencias, departamento de biología, universidad de tarapacá, arica.	9	286	1
**Bolivia**			
Instituto boliviano de biología de altura, la paz.	7	910	2

* Departments or areas belonging to the same institution.
¥ The number of documents and total link strength was obtained by the VosViewer tool.

Table 3. Top 50 journals with the highest number of publications in Scopus, 1980 - 2020

Journal	Publications	Citations	Journal	Publications	Citations
Journal of Applied Physiology	155	7937	British Journal Of Sports Medicine	13	461
High-altitude Medicine and Biology	338	6568	Frontiers In Physiology	37	441
New England Journal of Medicine	34	3453	Human Biology	11	420
Aerospace medicine and human performance	111	2343	Experimental Physiology	25	414
Medicine And Science In Sports And Exercise	31	1868	Journal Of Travel Medicine	27	410
Wilderness and Environmental Medicine	132	1769	JAMA The Journal Of The American Medical Association	14	409
Chest	49	1741	American Review Of Respiratory Disease	8	374
International Journal of Sports Medicine	49	1697	Respiration	12	362
Lancet	16	1458	Frontiers In Microbiology	10	329
American Journal Of Physical Anthropology	31	1429	Clinical Science	15	326
Circulation	16	1414	Western Journal Of Medicine	10	298
European Respiratory Journal	37	1199	Journal Of Wilderness Medicine	24	236
Respiratory Physiology and Neurobiology	44	1085	Sleep	11	225
Annals Of Internal Medicine	15	978	Sleep And Breathing	11	213
American Journal Of Respiratory And Critical Care Medicine	14	941	Medical Hypotheses	21	197
Respiration Physiology	22	907	Annals Of Human Biology	14	179
European Journal Of Applied Physiology	33	888	Postgraduate Medical Journal	13	172
American Journal Of Human Biology	32	721	Military Medicine	14	137
American Journal Of Physiology Regulatory Integrative And Comparative Physiology	15	676	Current Sports Medicine Reports	9	116
Placenta	12	676	International Journal Of Cardiology	10	104
Sports Medicine	13	642	Travel Medicine And Infectious Disease	9	101
Thorax	20	640	Journal Of The Royal Army Medical Corps	14	71
European Journal Of Applied Physiology And Occupational Physiology	20	588	Aerospace Medicine And Human Performance	25	68
Journal Of Cerebral Blood Flow And Metabolism	10	587	Wiener Medizinische Wochenschrift	14	67
British Medical Journal	8	541	Pulmonary Circulation	9	63
