# Supplementary material for: Adaptation and altitude sickness: A 40-year bibliometric analysis and collaborative networks
Source: Front Public Health. 2023 Mar 2;11:1069212. doi: 10.3389/fpubh.2023.1069212 (PMC10018125; doi:10.3389/fpubh.2023.1069212)

Supplementary material 3

Table 1. Analysis of the records obtained in Scopus using SciVal between, 2012 – 2020

Subject Area	Subcategory	Scholarly Output	Citations	Citations per Publication	Field-weighted Citation Impact
Medicine	-	729	7374	10.1	0.73
Medicine	Public Health, Environmental and Occupational Health	167	1301	7.8	0.7
Medicine	Physiology (medical)	102	1314	12.9	0.68
Medicine	General Medicine	85	492	5.8	0.51
Biochemistry, Genetics and Molecular Biology	-	243	2558	10.5	0.74
Biochemistry, Genetics and Molecular Biology	Physiology	179	1973	11	0.8
Biochemistry, Genetics and Molecular Biology	Genetics	34	279	8.2	0.53
Biochemistry, Genetics and Molecular Biology	Molecular Medicine	17	200	11.8	0.72
Health Professions	-	50	351	7	0.63
Health Professions	Physical Therapy, Sports Therapy and Rehabilitation	43	329	7.7	0.7
Health Professions	Medical Laboratory Technology	4	10	2.5	0.23
Health Professions	Pharmacy	1	0	0	0
Nursing	-	34	295	8.7	0.63
Nursing	Nutrition and Dietetics	25	171	6.8	0.47
Nursing	General Nursing	2	4	2	0.29
Nursing	Advanced and Specialized Nursing	2	102	51	2.83
Neuroscience	-	32	456	14.3	1.29
Neuroscience	General Neuroscience	13	251	19.3	2.18
Neuroscience	Neurology	11	145	13.2	0.89
Neuroscience	Neuroscience (miscellaneous)	5	13	2.6	0.2
Immunology and Microbiology	-	24	550	22.9	0.96
Immunology and Microbiology	Microbiology	10	386	38.6	1.1
Immunology and Microbiology	Immunology	5	76	15.2	1.09
Immunology and Microbiology	Virology	5	34	6.8	0.48
Pharmacology, Toxicology and Pharmaceutics	-	20	239	11.9	0.94
Pharmacology, Toxicology and Pharmaceutics	Pharmacology	10	76	7.6	0.7
Pharmacology, Toxicology and Pharmaceutics	Drug Discovery	7	174	24.9	1.74
Pharmacology, Toxicology and Pharmaceutics	General Pharmacology, Toxicology and Pharmaceutics	4	10	2.5	0.37
Psychology	-	6	89	14.8	0.96
Psychology	Clinical Psychology	5	62	12.4	0.75
Psychology	General Psychology	1	27	27	2.01
Psychology	Neuropsychology and Physiological Psychology	1	4	4	0.17
Dentistry	-	1	25	25	2.21
Dentistry	Oral Surgery	1	25	25	2.21

Table 2. International, national, and institutional collaboration, by scholarly output in Scopus using SciVal between, 2012 – 2020

Metric		Scholarly Output	Citations	Citations per Publication
International collaboration	33.40%	522	11264	21.6
Only national collaboration	34.90%	545	5651	10.4
Only institutional collaboration	22.10%	345	3534	10.2
Single authorship (no collaboration)	9.60%	150	1584	10.6

Table 3. top 20 authors with the most publications on adaptation and diseases at altitude in Scopus using SciVal between, 2012 – 2020

Author	Affiliation	Country/Region	Scholarly Output	Field-Weighted Citation Impact	Citation Count
Basnyat, Buddha	University of Oxford	United Kingdom	30	1.43	515
Ainslie, Philip N.	University of British Columbia	Canada	25	0.84	411
Bloch, Konrad Ernst	University of Zurich	Switzerland	22	2.14	694
Bar̈tsch, Peter D.	Heidelberg University	Germany	21	2.89	926
Swenson, Erik Richard	University of Washington	United States	19	3.12	756
Gao, Yuqi	Third Military Medical University	China	17	0.47	183
León-Velarde, Fabiola	Universidad Peruana Cayetano Heredia	Peru	17	1.34	388
Ulrich, Silvia	University of Zurich	Switzerland	16	2.05	438
Burtscher, Martin	University of Innsbruck	Austria	15	0.58	195
Furian, Michael	University of Zurich	Switzerland	15	2.09	412
Hackett, Peter H.	University of Colorado Anschutz Medical Campus	United States	15	3.62	668
Gore, Christopher John	Australian Institute of Sport	Australia	13	2.11	483
Julian, Colleen Glyde	University of Colorado Anschutz Medical Campus	United States	13	1.6	394
Latshang, Tsogyal Daniela	University of Zurich	Switzerland	13	0.86	188
Luks, Andrew Mark	University of Washington	United States	13	3.45	558
Moore, Lorna Grindlay	University of Colorado Anschutz Medical Campus	United States	13	1.62	453
Parati, Gianfranco	University of Milan - Bicocca	Italy	13	1.4	334
Scherrer, Urs	University of Bern	Switzerland	13	1.04	336
Hoiland, Ryan Leo	University of British Columbia	Canada	12	0.72	131
Mellor, Adrian J.	Leeds Beckett University	United Kingdom	12	0.61	119

Figure 1. Medical specialties with the most research in Scopus using SciVal between, 2012 – 2020

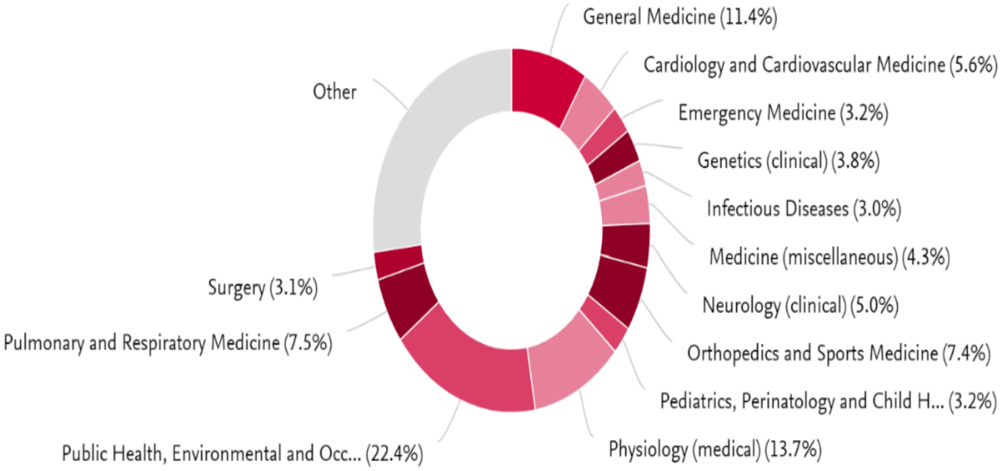

Figure 2. Top 25% of world topics by prominence in high altitude medicine in Scopus using SciVal between, 2012 – 2020

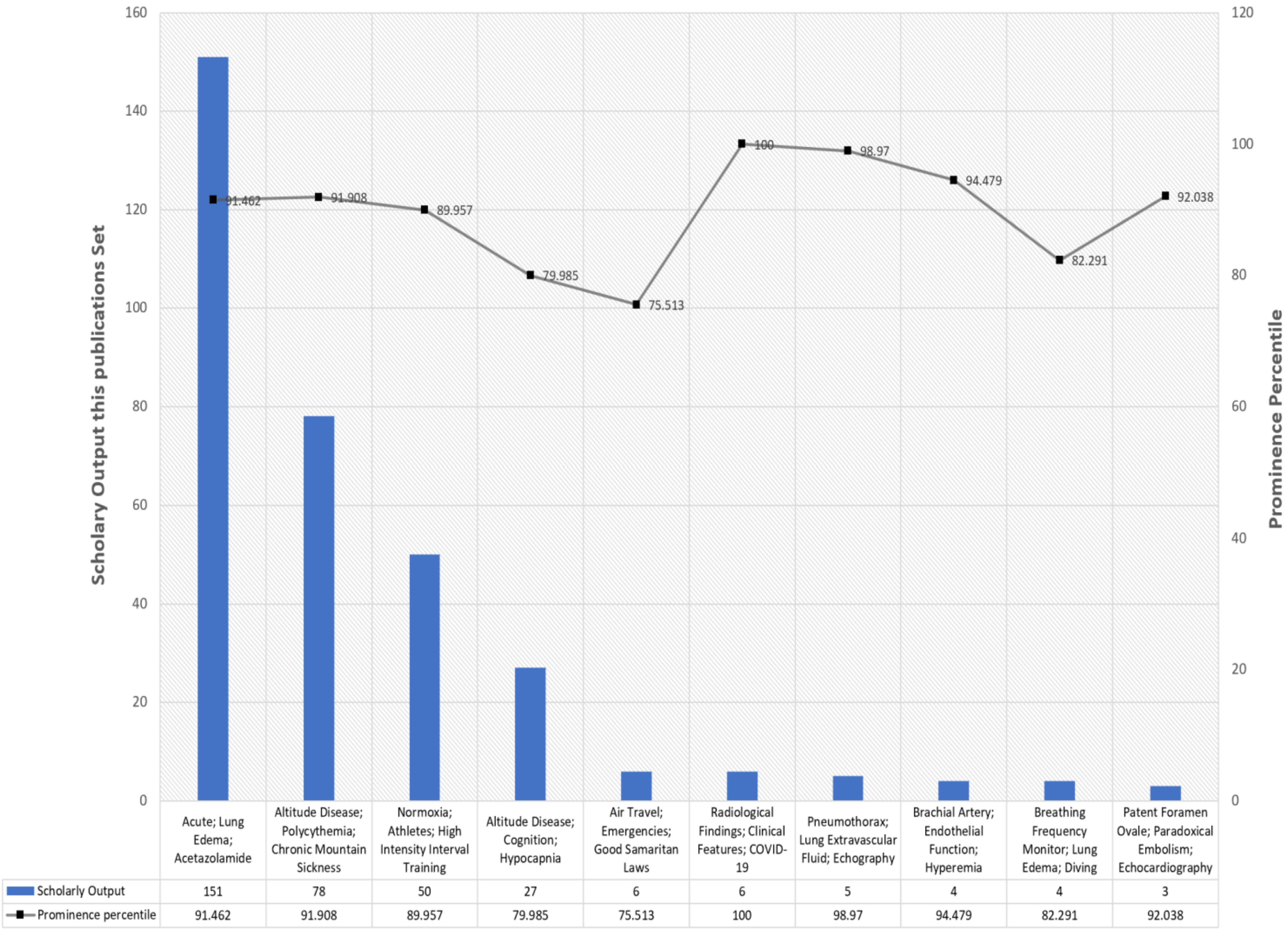

Supplement: Supplementary file 3 [file Data_Sheet_3.pdf]
